# Efficacy and safety analysis of a HER2-targeting antibody-drug conjugate combined with immune checkpoint inhibitors in solid tumors: a real-world study

**DOI:** 10.18632/aging.205382

**Published:** 2023-12-22

**Authors:** Yi-Xin Zhou, Jia-Ling Wang, Xiao-Li Mu, Ya-Juan Zhu, Ye Chen, Ji-Yan Liu

**Affiliations:** 1 Department of Biotherapy, Cancer Centre, West China Hospital, Sichuan University, Chengdu, China; 2 Division of Abdominal Tumor Multimodality Treatment, Department of Radiation Oncology, Cancer Center, West China Hospital, Sichuan University, Chengdu, China

**Keywords:** Disitamab Vedotin, immune checkpoint inhibitors, HER2, solid tumors

## Abstract

Background: Disitamab Vedotin is a novel antibody-drug conjugate (ADC) drug targeting HER2, which has shown a potential synergistic effect between Disitamab Vedotin and immune checkpoint inhibitors (ICIs). Therefore, we plan to conduct a retrospective real-world study to evaluate the efficacy and safety of Disitamab Vedotin monotherapy or combined with ICIs in the treatment of advanced or metastatic solid tumors.

Methods: This retrospective study involved patients with locally advanced or metastatic solid tumors who were treated with Disitamab Vedotin monotherapy or combined with ICIs at West China Hospital of Sichuan University from July 2019 to June 2023. The observation items included progression-free survival (PFS), overall survival (OS), objective response rate (ORR), disease control rate (DCR), and treatment-related adverse events (TRAEs).

Results: This study included 49 patients, out of which 34 patients were treated with Disitamab Vedotin plus ICIs and 15 patients received Disitamab Vedotin alone. In all patients, the median PFS was 10 months. The 6-month and 1-year OS rates were 91.1% and 82.3%, respectively. Eighteen (36.7%) patients achieved a partial response, and sixteen (32.7%) patients had stable disease. The combination therapy of Disitamab Vedotin plus ICIs showed a higher ORR (44.1% vs. 20.0%) and a longer median PFS (14 vs. 8 months) compared to Disitamab Vedotin alone. The median PFS for patients expressed with HER2 2+/3+ was 10 months and was not reached for patients expressed with HER2 0/1+. Grade 3–4 TRAEs occurred in 14.7% of patients who received the combination treatment and in 26.7% of patients who received Disitamab Vedotin alone.

Conclusions: Our study showed that Disitamab-Vedotin-based treatment, alone or in combination with ICIs, exerted considerable prognosis and good tolerance in patients with locally advanced or metastatic solid tumors, regardless of the HER2 expression levels. Whether combination therapy with ICIs provides greater therapeutic benefits compared to monotherapy needs to be further explored through randomized controlled trials.

## INTRODUCTION

Human epithelial growth factor receptor 2 (HER2) is a well-known oncogenic growth factor from the epidermal growth factor receptor (EGFR) family. HER2 overexpression results in the autophosphorylation of tyrosine residues within the cytoplasmic domain of the heterodimer and leads to cell proliferation and tumorigenesis [1]. The overexpression of HER2 has been identified in multiple cancers including breast, gastric, urothelial, and colorectal cancer [2–5], and has also been recognized to be correlated with metastasis and poor prognosis of these tumors [6, 7]. To date, HER2-targeting therapy has dramatically improved the prognosis of patients with locally advanced or metastatic cancers [8]. Recently, HER2-targeting antibody-drug conjugates (ADCs), such as TDM-1 (trastuzumab deruxtecan), showed considerable efficacy in metastatic patients for post-line therapy [9].

Disitamab Vedotin is a novel ADC drug that contains a humanized HER2-targeting antibody hertuzumab and a monomethyl auristatin E (MMAE) conjugated by a cleavable linker. Disitamab Vedotin exerts its anti-tumor effects through two main pathways: firstly, by inhibiting downstream signaling pathways activated by HER2, thereby interfering with cell transcription, growth, and proliferation; secondly, by interfering with microtubule formation through the small molecule MMAE, which mainly results in cell cycle arrest. In addition, *in vitro* studies have demonstrated that Disitamab Vedotin exhibits antibody-dependent cell-mediated cytotoxicity (ADCC) effects against HER2-overexpressing cancer cells, thereby exerting a tumor-inhibitory effect [10]. Compared with previous HER2-targeting antibodies such as trastuzumab, hertuzumab has a higher affinity to HER2 and a more effective ADCC activity [11]. Furthermore, the cleavable linker of Disitamab Vedotin enables anti-tumor activity towards HER2-negative tumor cells through the bystander effect, which might overcome resistance caused by the heterogeneity of HER2 expression levels among tumor cells [10, 12]. A Phase II study of Disitamab Vedotin in patients with locally advanced or metastatic urothelial carcinoma demonstrated an ideal efficacy by reporting an ORR of 51.2% and a median progression-free survival (PFS) of 6.9 months [10]. Another Phase II study of Disitamab Vedotin in patients with gastric cancer and conferring 2 prior therapies indicated an ORR of 24.4% and a median PFS of 4.1 months [13]. Currently, the approval of Disitamab Vedotin in China is limited to the treatment of locally advanced or metastatic urothelial carcinoma with HER2 overexpression that has received prior platinum-containing chemotherapy and locally advanced or metastatic gastric cancer with HER2 overexpression that has undergone at least two prior systemic therapies. However, there is still a lack of clinical studies on the use of Disitamab vedotin in pan-tumor treatment.

Immunotherapy employs monoclonal antibodies to target immune checkpoint inhibitors (ICIs) produced by tumors, which inhibit T cells from recognizing and destroying cancer cells [14]. ICIs are the standard post-line therapies for many advanced or metastatic cancers such as urothelial cancer [15]. In the Disitamab Vedotin-C014 trial, Disitamab Vedotin combined with toripalimab treating urothelial cancer showed promising efficacy with an ORR of 75% of all patients and 80% of previously untreated patients in the first-line setting [16]. Preclinical studies also suggested that the combination of Disitamab Vedotin and ICIs had remarkable effects and showed long-lasting immune protection in a humanized HER2+ murine breast cancer model [17]. Therefore, the combination of Disitamab Vedotin and ICIs might have therapeutic potential.

There is currently no real-world study supporting the use of Disitamab Vedotin alone or in combination with ICIs for treating advanced or metastatic solid cancers except urothelial carcinoma. To address this, we conducted this study on patients with locally advanced or metastatic cancers who were treated with Disitamab Vedotin alone or in combination with ICIs to evaluate the safety and effectiveness of these therapies.

## MATERIALS AND METHODS

### Study design and patients

**Figure 1:**
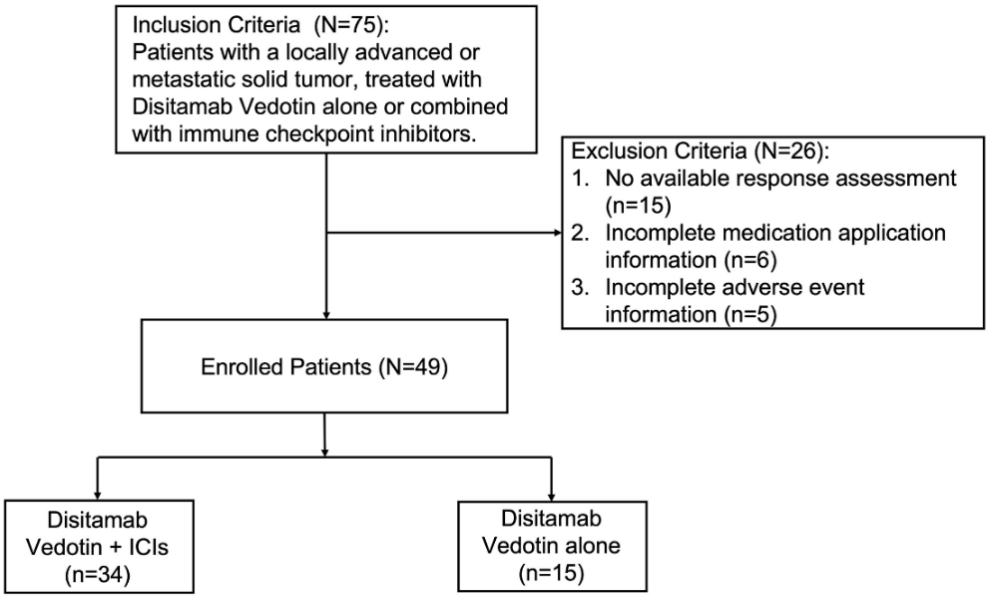
Flow diagram of selecting patients.

Data of 75 cases regarding initially diagnosed with advanced or metastatic solid tumors and treated with Disitamab Vedotin alone or combined with ICIs was obtained from the Cancer Centre, West China Hospital of Sichuan University, corresponding to the period from July 2019 to June 2023. Patients without available clinicopathologic characteristics were excluded. The detailed data selection process and criteria are shown in Figure 1. The study protocol was approved by the ethical committee of the West China Hospital of Sichuan University.

### Data collection

The extracted variables included demographics, disease characteristics, treatments, laboratory test results, imaging scans, and treatment-related adverse events (TRAE). All patients were treated with Disitamab Vedotin until disease progression, intolerable TRAE, or death. The application of ICI administration was based on the judgment of the physician. HER2 expression is primarily detected using immunohistochemistry (IHC). The IHC scores are evaluated based on the guidelines specified for HER2 testing in breast cancer [18].

### Outcomes definition

The observation items included progression-free survival (PFS), overall survival (OS), objective response rate (ORR), disease control rate (DCR), and treatment-related adverse events (TRAEs). The PFS and OS were measured as months from the initiation of therapy until progression or death. The objective response was determined as the best response sustained from the first treatment until the end of the follow-up. The performance was evaluated using Response Evaluation Criteria in Solid Tumors (RECIST) version 1.1. The enhanced CT scans were routinely performed at baseline and every 6-8 weeks. We analyzed the disease control rate (DCR), objective response rate (ORR), and treatment-related adverse events (TRAEs). DCR was determined by calculating the percentage of patients who achieved complete response (CR), partial response (PR), or stable disease (SD). ORR was calculated as the percentage of patients who achieved CR or PR. The swimmer plot was performed to show the time points of vital clinical events of each patient. The TRAEs were evaluated according to the Common Terminology Criteria for Adverse Events (CTRAE) version 5.0. The relation of each TRAEs with Disitamab Vedotin and ICIs was evaluated according to physicians’ experience.

### Statistical analysis

The SPSS 25.0 (IBM, Armonk, NY, USA) and the R project (version 4.1.2) were applied for statistical analysis. The Chi-squared test was used to compare baseline characteristics between the two groups. Descriptive statistics were used to analyze demographic characteristics, treatment administrations, laboratory results, and TRAEs. The Kaplan-Meier and the Log-rank test method was used to evaluate the survival outcomes of patients. Multivariate survival analyses were performed with the Cox proportional hazards model, and Hazard ratios (HRs) were computed with 95% confidence intervals (CIs). All statistical tests were two-sided, and P < 0.05 was considered statistically significant.

### Data availability

The datasets used and/or analyzed during the current study are available from the corresponding author upon reasonable request.

## RESULTS

### Patients’ characteristics

**Figure 2:**
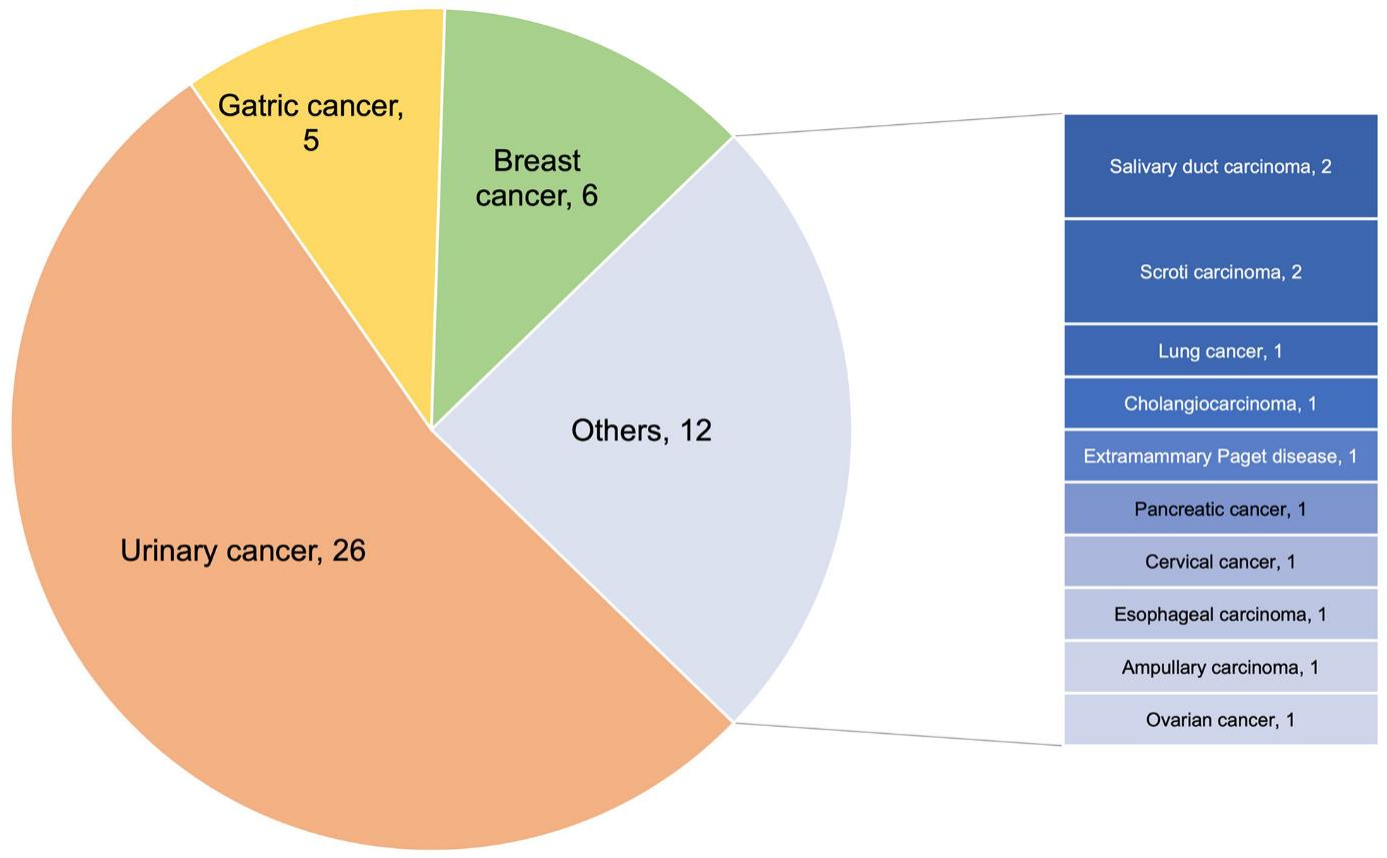
Cancer types of enrolled patients.

A total of 75 patients treated with Disitamab-based therapy were included. By the end of the follow-up period, 15 patients were still being treated, and the time for the first efficacy evaluation had not arrived (Figure 1). We have excluded 6 patients without complete medication application information, and 5 without complete adverse event information. The final cohort consisted of 49 patients, with 34 patients receiving Disitamab Vedotin combined with ICIs treatment and 15 patients receiving Disitamab Vedotin alone (Figure 1). The detailed ICI treatments were listed in Supplementary Table 1, including PD-1, PD-L1, and PD-1+CTL-4 inhibitors. Our study reported a median follow-up duration of 8 months (range: 1-28 months) for patients treated with Disitamab Vedotin in combination with ICIs, and 9 months (range: 1-15 months) for those treated with Disitamab Vedotin monotherapy. More than half of the patients were females. The distribution of cancer types among the patients is presented in Figure 2. More than half of the patients in this study were diagnosed with urinary cancer, followed by breast cancer, gastric cancer and other kinds of cancer, such as salivary duct carcinoma, scorti carcinoma. The patients aged from 28 to 86 years old, with a median age of 59 (Table 1). HER2 expression was positive (IHC 3+, or 2+) in 55.9% of patients treated with Disitamab Vedotin plus ICIs and in 86.7% of patients treated with Disitamab Vedotin alone. Eight HER-2 negative patients were all treated with combination therapy, which was a clinical decision made by the treating physicians based on their judgment. Most patients had multiple metastases before the application of Disitamab Vedotin or Disitamab Vedotin in combination with ICIs. Two-thirds of patients have received at least first-line treatment in the past. Additionally, approximately one-third of patients received Disitamab Vedotin alone or in combination with ICIs as the first-line therapy, as they were intolerant to platinum-based chemotherapy or refused to undergo platinum-containing regimens. The median treatment cycles for patients treated with Disitamab Vedotin combined with ICIs and Disitamab Vedotin monotherapy were 4 (range 2-15) and 2 (range 1-7), respectively. No statistically significant difference in patients’ characteristics was observed between the two groups. The characteristics of patients with other tumors rather than urinary cancer were listed in the Supplementary Table 3.

**Table 1:** The demographic and clinical characteristics of eligible patients treated with Disitamab Vedotin combined with ICIs and Disitamab Vedotin alone.

**Variables**	**Disitamab Vedotin+ICIs N=34, n (%)**	**Disitamab Vedotin alone N=15, n (%)**	**P-values**
**Median Age (range)**	59 (28-86)	60 (38-85)	
**Sex**			0.164
Male	18 (52.9)	4 (26.7)	
Female	16 (47.1)	11 (73.3)	
**HER2 expression levels**			0.085
0	3 (8.8)	0 (0.0)	
1+	5 (14.7)	0 (0.0)	
2+	14 (41.2)	6 (40.0)	
3+	5 (14.7)	7 (46.7)	
Unknown	7 (20.6)	2 (13.3)	
**Pathology**			0.192
Urothelial carcinoma	18 (52.9)	7 (46.7)	
Adenocarcinoma	10 (29.4)	2 (13.3)	
Others	6 (17.6)	6 (40.0)	
**Cancer types**			0.775
Urinary cancer	19 (55.9)	7 (46.7)	
Others	15 (44.1)	8 (53.3)	
**Metastasis**			
No	6 (17.6)	5 (33.3)	0.400
Yes	28 (82.4)	10 (66.7)	
**Metastatic sites**			
Bone	9 (26.5)	6 (40.0)	0.541
Brain	1 (2.9)	3 (20.0)	0.149
Liver	7 (20.6)	6 (40.0)	0.286
Lung	9 (26.5)	4 (26.7)	1.000
Lymph nodes	15 (44.1)	5 (33.3)	0.695
Others	9 (26.5)	1 (6.7)	0.230
**Prior Surgery**			0.220
No	12 (35.3)	2 (13.3)	
Yes	22 (64.7)	13 (86.7)	
**Prior Chemotherapy**			0.252
No	19 (55.9)	5 (33.3)	
Yes	15 (44.1)	10 (66.7)	
**Prior Radiotherapy**			0.883
No	25 (73.5)	10 (66.7)	
Yes	9 (26.5)	5 (33.3)	
**Prior Targeted Therapy**			0.399
No	24 (70.6)	8 (53.3)	
Yes	10 (29.4)	7 (46.7)	
**Prior Immunotherapy**			
No	27 (79.4)	10 (66.7)	0.551
Yes	7 (20.6)	5 (33.3)	
**Number of prior treatment lines**			0.241
0	12 (35.3)	5 (33.3)	
1	19 (55.9)	6 (40.0)	
>=2	3 (8.8)	4 (26.7)	
**Dosage of RC48**			1.000
120mg, d1, q2w	28 (82.4)	12 (80.0)	
<120mg	6 (17.6)	3 (20.0)	
**Median number of treatment cycles (range)**	4 (2-18)	2 (1-15)	

### Efficacy assessment

**Figure 3:**
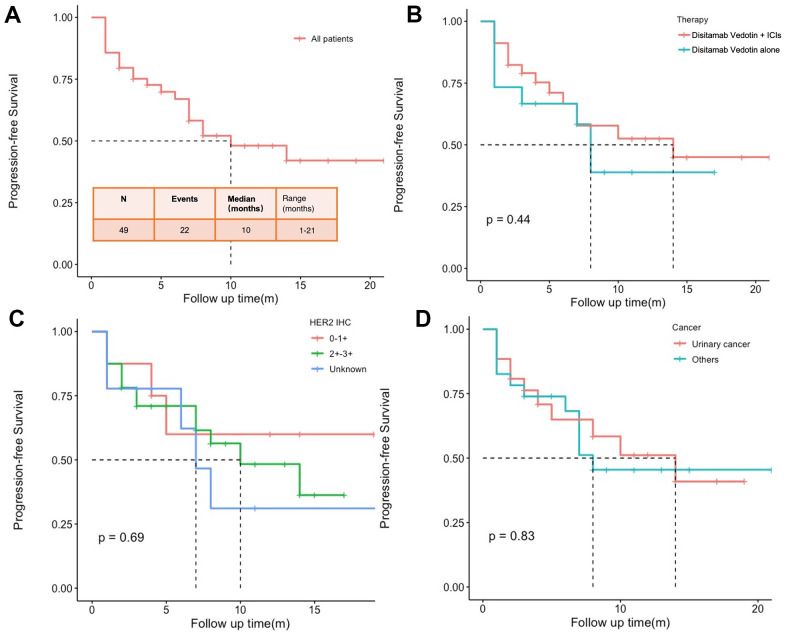
Progression-free survival (PFS) of all patients (**A**), patients treated with Disitamab Vedotin combined with ICIs and Disitamab Vedotin alone (**B**), patients with different expression levels of HER2 (**C**), and patients with urinary cancer and other types of cancer (**D**).

At the end of the follow-up period, disease progression was observed in 22 patients and 8 patients had died. All patients’ median PFS was 10.0 (range 1-21) months (Figure 3A). The 6-month and 1-year OS rates were 91.1% and 82.3%, separately. The median PFS for patients who received Disitamab Vedotin in combination with ICIs and those treated with Disitamab Vedotin alone was 14 (range 1-21) and 8 (range 1-17) months, respectively (Figure 3B). The median PFS for patients expressed with HER2 2+/3+ was 10 (range 1-17) months and was not reached for patients expressed with HER2 0/1+ (Figure 3C). All eight patients with HER2 0/1+ were all treated with Disitamab combined with ICIs. The median PFS of 14 (range 1-18) and 8 (range 1-21) months was achieved among patients with urinary cancer and other types of cancer (Figure 3D). A subgroup survival analysis was conducted specifically for urinary cancer. The study found that the median PFS for patients with urinary cancer who received treatment with Disitamab Vedotin combined with ICIs was 10 (range 1-17) months (Supplementary Figure 1A). On the other hand, the median PFS was not reached among patients who were treated with Disitamab Vedotin alone. Among the patients with UC (n=26), 5 exhibited HER2 0/1+, while 18 presented HER2 2/3+. Given the limited sample size, a direct comparison between the combined therapy and monotherapy in the HER2 0/1+ patients was not feasible. However, such a comparison was conducted within the HER2 2/3+ population. The results indicated that the median PFS for the combined group and the monotherapy group were 11 and 8 months, respectively (P=0.67). The median PFS for patients with other cancers rather than urinary cancer were 11 (range 1-21) and 8 (range 1-9) months, separately (Supplementary Figure 1B). The Cox analysis of PFS in the stratified subgroup did not reveal a statistically significant difference between the efficacy of Disitamab Vedotin monotherapy and the combination of Disitamab Vedotin with ICIs (Supplementary Figure 2).

**Figure 4:**
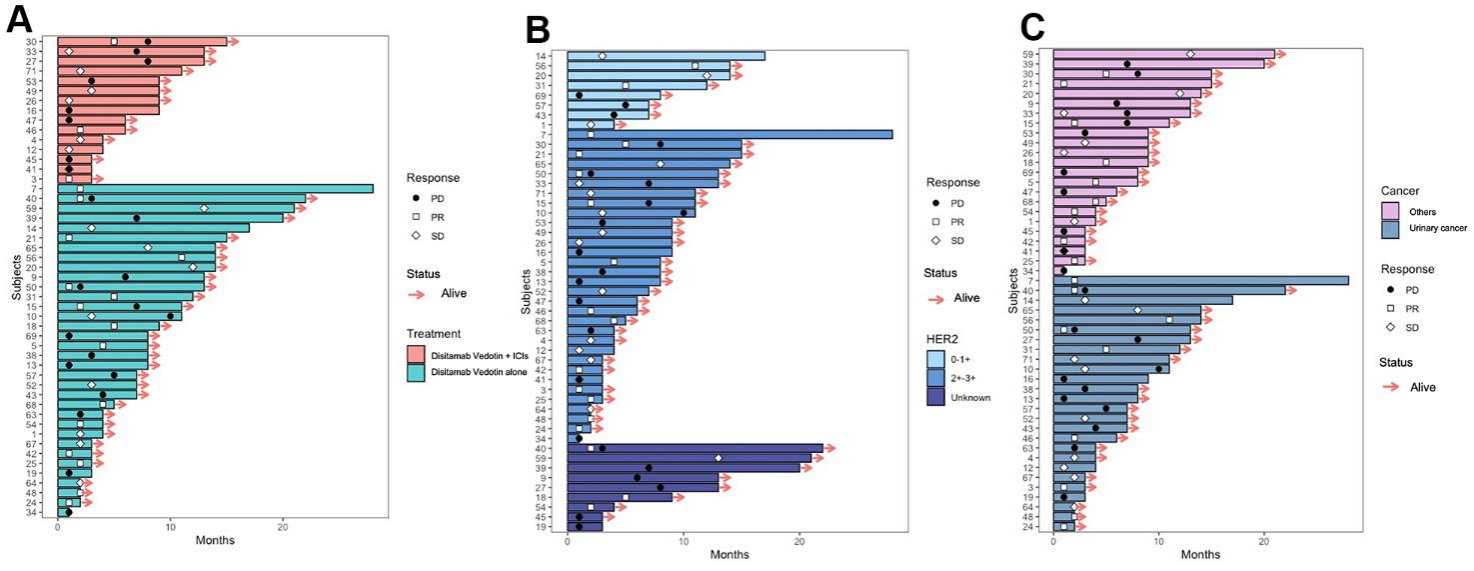
Swimmer plot of the patients receiving Disitamab Vedotin alone and combined with ICI (**A**). Swimmer plot of the patients with different expression levels of HER2 (**B**). Swimmer plot of the patients with urinary cancer and other cancers (**C**).

The swimmer plot for all patients is shown in Figure 4. Although many patients have shown clinical efficacy of the treatments, most of them eventually obtained disease progression. Of all the patients in the study, 18 (36.7%) achieved the best response to PR, while 15 (30.6%) only experienced PD (Table 2). The ORR and DCR for all patients were 36.7% (95% CI 23.8-51.7%) and 69.4%, (95% CI 55.5-80.5%) respectively (Table 2). The ORRs for patients treated with Disitamab Vedotin and ICIs and Disitamab alone were 44.1% (95% CI 27.6-61.9%) and 20.0% (95% CI 5.3-48.6%), separately. Patients expressed with HER2 2-3+ achieved an ORR and DCR of 40.6% (95% CI 24.2-59.2%) and 78.1% (95% CI 59.6-90.1%), separately. While patients expressed with HER2 0-1+ only achieved an ORR and DCR of 25.0% (95% CI 4.5-64.4%) and 62.5% (95% CI 30.6-86.3%), respectively. Despite patients with urinary cancer, other patients with varied cancer types achieved an ORR and DCR of 39.1% (95% CI 20.5-61.2) and 69.6% (95% CI 47.0-85.6%), separately. Among patients with urinary cancer, 9 (34.6%) of them achieved PR, including 7 (36.8%) patients treated with Disitamab Vedotin combined with ICIs and 2 (28.6%) patients treated with Disitamab Vedotin alone (Supplementary Table 2). The ORRs of patients with urinary cancer treated with Disitamab Vedotin and ICIs and Disitamab monotherapy were 36.8% (95% CI 17.2-61.4%) and 28.6% (95% CI 5.1-69.7%), respectively. Regarding patients with rare tumors rather than urinary cancer, the detailed information was listed in the Supplementary Table 3.

**Table 2:** Best overall tumor responses in the full-analysis and efficacy-evaluable populations.

**Populations**	**Total no.**	**PD, no. (%)**	**SD, no. (%)**	**PR, no. (%)**	**ORR (95% CI)**	**DCR (95% CI)**
**Treatment**						
Disitamab Vedotin + ICIs	34	10 (29.4)	9 (26.5)	15 (44.1)	44.1 (27.6-61.9)	70.6 (52.3-84.3)
Disitamab Vedotin alone	15	5 (33.3)	7 (46.7)	3 (20.0)	20.0 (5.3-48.6)	66.7 (38.7-87.0)
**HER2 expression**						
0-1+	8	3 (37.5)	3 (37.5)	2 (25.0)	25.0 (4.5-64.4)	62.5 (30.6-86.3)
2-3+	32	7 (21.9)	12 (37.5)	13 (40.6)	40.6 (24.2-59.2)	78.1 (59.6-90.1)
Unknown	9	5 (55.6)	1 (11.1)	3 (33.3)	33.3 (9.0-69.1)	44.4 (15.3-77.3)
**Cancer type**						
Urinary cancer	26	8 (30.8)	9 (34.6)	9 (34.6)	34.6 (18.0-55.6)	69.2 (48.1-84.9)
Others	23	7 (30.4)	7 (30.4)	9 (39.1)	39.1 (20.5-61.2)	69.6 (47.0-85.6)
**All populations**	**49**	**15 (30.6)**	**16 (32.7)**	**18 (36.7)**	**36.7 (23.8-51.7)**	**69.4 (55.5-80.5)**

Multivariate Cox proportional hazard analysis was applied to evaluate the potential effects of clinical variables on PFS (Supplementary Figure 3). Females were found to have a poorer prognosis than males. patients treated with Disitamab Vedotin as a second or third-line therapy had a worse prognosis than those who received it as a first-line treatment. None of the other variables were found to be independent prognostic factors of PFS.

### Safety profile

The TRAEs in patients treated with Disitamab Vedotin in combination with ICIs or Disitamab Vedotin alone are listed in Table 3. Grade 3–4 TRAEs occurred in 5 (14.7%) patients who received the combination treatment e, and in 4 (26.7%) patients who received solely Disitamab Vedotin. No treatment-related death was observed in either two cohorts. The most common TRAE were anemia (in 10 [32.4%] of patients treated with Disitamab Vedotin combined with ICIs, and in 3 [20%] of patients treated with Disitamab Vedotin alone), elevated aspartate transaminase (AST) /alanine aminotransferase (ALT), hypoalbuminemia, and nausea. And compared with the Disitamab Vedotin-alone group, the incidence of overall TRAE or grade 3-4 TRAE in the Disitamab Vedotin combined with ICIs group has no significant statistical difference (85.3% vs. 86.7%, P = 1.00; 14.7% vs 26.7%, P=0.55).

**Table 3:** Summary of the adverse events.

**Events, n (%)**	**Patients (n=49)**		**Patients treated with Disitamab Vedotin + ICIs (n=34)**		**Patients treated with Disitamab Vedotin alone (n=15)**	
	**Any grades**	**Grade 3-4**	**Any grades**	**Grade 3-4**	**Any grades**	**Grade 3-4**
**Any adverse events**	**42 (85.7)**	**9 (18.4)**	**29 (85.3)**	**5 (14.7)**	**13 (86.7)**	**4 (26.7)**
**Haematology**						
Anemia	14 (28.6)	3 (6.1)	11 (32.4)	3 (8.8)	3 (20.0)	0 (0.0)
Leukopenia	4 (8.2)	3 (6.1)	2 (5.9)	1 (2.9)	2 (13.3)	2 (13.3)
Neutropenia	8 (16.3)	2 (4.1)	6 (17.6)	1 (2.9)	2 (13.3)	1 (6.7)
Thrombocytopenia	5 (10.2)	3 (6.1)	3 (8.8)	1 (2.9)	2 (13.3)	2 (13.3)
**Renal and Liver function**						
Elevated ALT	8 (16.3)	1 (2.0)	5 (14.7)	1 (2.9)	3 (20.0)	0 (0.0)
Elevated AST	12 (24.5)	1 (2.0)	7 (20.6)	1 (2.9)	5 (33.3)	0 (0.0)
Hypoalbuminemia	18 (36.7)	3 (6.1)	14 (41.2)	3 (8.8)	4 (26.7)	0 (0.0)
**Symptoms**						
Hypesthesia	3 (6.1)	0 (0.0)	2 (5.9)	0 (0.0)	1 (6.7)	0 (0.0)
Fatigue	1 (2.0)	0 (0.0)	0 (0.0)	0 (0.0)	1 (6.7)	0 (0.0)
Hair loss	9 (18.4)	2 (4.1)	4 (11.8)	0 (0.0)	5 (33.3)	2 (13.3)
Pruritus	7 (14.3)	1 (2.0)	6 (17.6)	1 (2.9)	1 (6.7)	0 (0.0)
Rash	3 (6.1)	0 (0.0)	3 (8.8)	0 (0.0)	0 (0.0)	0 (0.0)
Nausea	8 (16.3)	0 (0.0)	6 (17.6)	0 (0.0)	2 (13.3)	0 (0.0)
Diarrhea	2 (4.1)	0 (0.0)	1 (2.9)	0 (0.0)	1 (6.7)	0 (0.0)
Vomit	3 (6.1)	0 (0.0)	3 (8.8)	0 (0.0)	0 (0.0)	0 (0.0)
Constipation	2 (4.1)	0 (0.0)	0 (0.0)	0 (0.0)	2 (13.3)	0 (0.0)
Loss of appetite	4 (8.2)	0 (0.0)	2 (5.9)	0 (0.0)	2 (13.3)	0 (0.0)
Pneumonia	1 (2.0)	1 (2.0)	1 (2.9)	1 (2.9)	0 (0.0)	0 (0.0)
Hypothyroidism	11 (22.4)	0 (0.0)	8 (23.5)	0 (0.0)	3 (20.0)	0 (0.0)

## DISCUSSION

These results showed the promising therapeutic benefits of Disitamab Vedotin alone or in combination with ICIs in patients with locally advanced or metastatic solid tumors. The combination therapy of Disitamab Vedotin plus ICIs showed a higher ORR and longer PFS. The safety analysis of TRAE further assured that it is relatively safe to use Disitamab Vedotin alone or combined with ICIs in patients. Furthermore, our results indicated considerable clinical efficacy in patients with HER2-negative (0-1+). Our study on Disitamab Vedotin for advanced or metastatic solid tumors identifies potential risks and subgroups of patients who may benefit from its use.

The overexpression of HER2 has been recognized as a poor prognostic factor for multiple cancers, including urothelial cancer, breast cancer, and gastric cancer [19–21]. HER2-targeting therapy is a potential strategy for multiple advanced or metastatic tumors that overexpressed HER2. As the first humanized HER2-targeting antibody, trastuzumab has been investigated and recognized as a standard therapy for HER2-positive metastatic breast cancer and achieved excellent therapeutic benefits in clinical applications [22, 23]. However, although some case reports have suggested that trastuzumab and chemotherapy can lead to long-lasting remission in resistant patients, some phase II RCTs failed to demonstrate any benefits of combining trastuzumab with chemotherapy [24–26].

Several ADC drugs based on HER2-targeting antibodies have been investigated and exerted excellent clinical benefits, including TDM-1, trastuzumab duocarmazine, and Disitamab Vedotin. A phase II study (KAMELEON) of single-agent T-DM1 in 69 patients with HER2-positive advanced urothelial bladder cancer showed an ORR of 38.5% (95% CI, 16.6 to 64.5) [27]. A Phase III clinical trial of treating patients with residual invasive HER2-positive breast cancer using TDM-1 or trastuzumab indicated that the disease-free survival was significantly higher in the T-DM1 group than in the trastuzumab group (HR, 0.5; 95% CI, 0.4 to 0.6; P<0.001) [28]. In the phase I clinical trial of treating locally advanced and metastatic solid tumors and HER2-expressing breast cancer with trastuzumab duocarmazine, an ORR of 6% was achieved in patients with gastric cancer, 25% in patients with urothelial cancer, and 39% in patients with endometrial cancer [29]. A prospective study of Disitamab Vedotin in patients with urothelial cancer showed an ORR of 51.2% and a median PFS of 6.9 months [10]. In our research, treating patients with solid tumors with Disitamab Vedotin alone achieved an ORR of 20% and a median PFS of 10 months. Among patients with urinary cancer, Disitamab Vedotin alone achieved an ORR of 28.6%, and the median PFS was not reached.

Furthermore, the combination of HER2-based ADC and immunotherapy also showed some ideal results. A preclinical study reported promising outcomes of combining Disitamab Vedotin and ICIs in a humanized HER2+ murine breast cancer model [17]. A Phase I multicenter clinical trial of trastuzumab deruxtecan in combination with nivolumab reported an ORR of 36.7% and a PFS of 6.9 months [30]. In a prospective study, the combination of Disitamab Vedotin and toripalimab demonstrated an ORR of 75%. [16]. A retrospective study was conducted to examine the treatment of urothelial cancer patients with Disitamab Vedotin alone or in combination with ICIs. The study found that the ORR was 38.9% and the median PFS was 8.5 months [31]. Compared with our study, an ORR of 44.1% and a median PFS of 10 months were achieved among patients with advanced or metastatic solid tumors treated with Disitamab Vedotin in combination with ICIs, including 36.8% and 10 months in patients with urothelial cancer. Earlier studies suggested that the median PFS of patients treated with a combination of Disitamab Vedotin and ICIs was longer compared to those who received Disitamab Vedotin monotherapy [31]. However, this trend was not explicitly observed in our cohort of urothelial cancer patients, although it remained consistent across all patients (14 months vs. 8 months). Nonetheless, no statistical significance was observed in either group. In order to gain more insights, future studies with larger sample sizes could be conducted to explore the optimal combination strategy, potential benefits, and specific patient populations that may derive the greatest benefits from these treatments. To date, a Phase III study of applying Disitamab Vedotin combined with toripalimab for first-line treatment of metastatic urothelial cancer is still undergoing.

Despite the assured therapeutic effects of Disitamab Vedotin in patients expressed with HER2-positive, it is believed that Disitamab Vedotin also has anti-tumor effects on tumors with heterogeneous antigen expression due to the bystander effect. Based on its cleavable linker, this bystander effect appears to be unique to Disitamab Vedotin and was not observed in TDM-1 [12, 32]. An analysis of HER2 status in molecular subtypes of 139 cases with urothelial carcinoma indicated 97 (65%) had IHC scores of 0 or 1+, 38 (26%) scored 2+, and 13 (9%) scored 3+ [33]. Regarding gastric cancer, a study of 122 patients reported 13.9% of patients expressed HER2-negative [34]. Although many patients have low HER2 levels, no HER2-targeted therapies are currently approved for the population with an IHC score of 0 or 1+. A phase Ib/II clinical trial (Disitamab Vedotin-C014) of 39 patients with urothelial cancer recently demonstrated a considerable ORR of 100% for patients with HER2 3+, 77.8% for HER2 2+, 66.7% for HER2 1+, and 50% for HER2 0, respectively [16]. Our research showed an ORR of 33.3% for urinary cancer patients treated with Disitamab Vedotin combined with ICIs with HER2 2-3+ and 40.0% for HER2 0-1+. Among all types of solid tumors, an ORR of 40.6% was observed in patients with HER2 2-3+, and 37.5% for HER2 0-1+. The promising activity of Disitamab Vedotin in combination with ICIs in HER2-positive cancer, even in tumors with low HER2 expression levels, makes it an appealing treatment for further investigation. This could potentially expand the targeted population of interest. However, all the patients with HER2 0-1+ underwent Disitamab Vedotin combined with ICIs, instead of Disitamab Vedotin monotherapy. Therefore, the potential therapeutic outcomes of treating HER2-negative patients with only Disitamab Vedotin require future research.

The safety profile of Disitamab Vedotin alone or in combination with ICI was similar in the current study. The study’s most frequently observed treatment-related adverse events were related to hematological and renal or liver toxicities, similar to previous studies [35]. The incidence of ≥ grade 3 AEs (26.7%) among patients treated with Disitamab Vedotin alone, was lower than those of the similar drug T-DM1 (60%) [36], and also lower than a previous clinical trial based on treating patients with solid tumors with Disitamab Vedotin alone (59.6%) [35], but higher than another retrospective research (11.1%) [31]. Regarding patients treated with Disitamab Vedotin combined with ICIs, the incidence of ≥ grade 3 TRAEs (14.7%) was lower than in previous studies based on Disitamab Vedotin combined with ICIs (27.8%) [31]. The variation in TRAE incidence may be attributed to the varying events and populations that different studies focused on. In our study, we found that patients with locally advanced or metastatic tumors tolerated Disitamab Vedotin alone or in combination with immune checkpoint inhibitors (ICIs) well. These findings are consistent with previous research and provide further support for the good tolerability of these treatments.

This study has limitations, including its retrospective nature and the heterogeneity in patients’ characteristics and therapeutic factors, which factors could potentially introduce bias into the study results. Additionally, despite our study included patients with multiple cancers to explore possible indications, the sample size of each cancer type was limited. Therefore, further randomized controlled trial research based on multiple tumors with a larger sample size is needed to assess the treatment of Disitamab Vedotin alone or in combination with ICIs.

To our knowledge, this study represents the first comprehensive analysis of the efficacy of Disitamab-Vedotin-based treatment in patients with locally advanced or metastatic solid tumors across a range of tumor types. Our findings indicate that Disitamab Vedotin alone or in combination with ICIs has potent anti-tumor effects and good tolerance, regardless of the HER2 expression levels. Randomized controlled trials are warranted to further evaluate the efficacy of Disitamab Vedotin as monotherapy versus in combination with ICIs in solid tumors.

## Supplementary Material

Supplementary Figures

Supplementary Tables
