# Supplementary material for: Efficacy and safety analysis of a HER2-targeting antibody-drug conjugate combined with immune checkpoint inhibitors in solid tumors: a real-world study
Source: Aging (Albany NY). 2023 Dec 22;15(24):15473–88. doi: 10.18632/aging.205382 (PMC10781476; doi:10.18632/aging.205382)
Supplement: Supplementary Figures [file aging-15-205382-s001.pdf]

SUPPLEMENTARY FIGURES

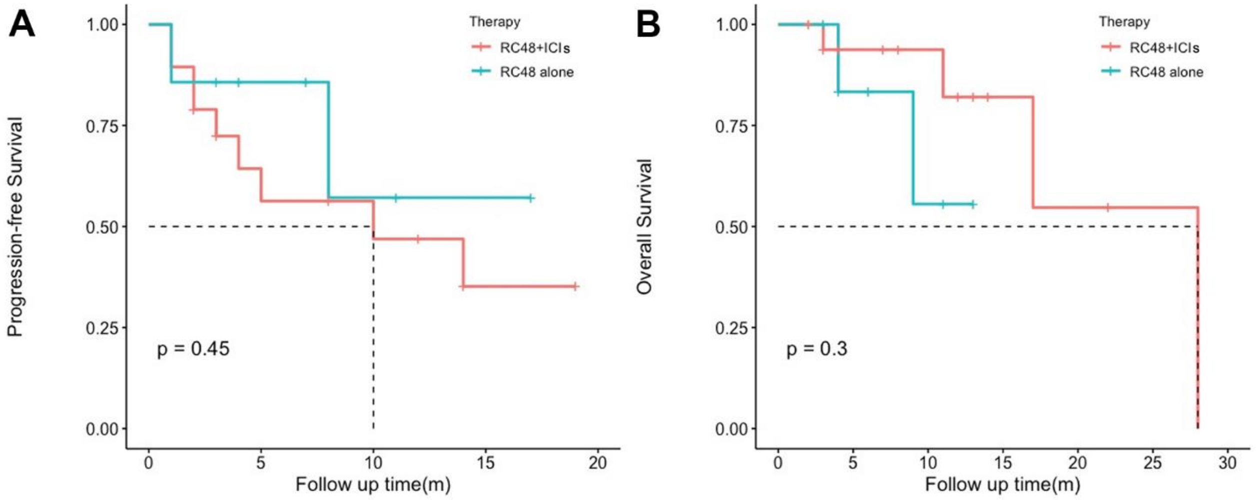

Supplementary Figure 1. Progression-free survival (PFS) of patients with urinary cancer (**A**) and other cancers (**B**).

Variables	Disitamab Vedotin+ICIs	Disitamab Vedotin alone	P value	HR (95% CI)
All patients	34	15	0.45	1.39 (0.58-3.34)
Age				
<60	17	8	0.24	2.06 (0.63-6.78)
>=60	17	7	0.49	1.96 (0.31-12.57)
Sex				
Male	18	4	0.58	0.60 (0.07-4.76)
Female	16	11	0.91	0.91 (0.32-2.57)
Pathology				
Urothelial Carcinoma	18	7	0.44	1.84 (0.39-8.78)
Adenocarcinoma	10	2	0.31	0.31 (0.03-3.01)
Others	6	6	0.17	0.22 (0.02-1.92)
Metastasis				
Yes	6	5	0.78	1.42 (0.13-15.89)
No	28	10	0.74	0.74 (0.28-1.96)
Prior surgery				
No	12	2	0.78	1.37 (0.16-11.51)
Yes	22	13	0.2	2.04 (0.68-6.07)
Prior chemotherapy				
No	19	5	0.76	0.72 (0.09-5.98)
Yes	15	10	0.71	1.21 (0.44-3.36)
Prior radiotherapy				
No	25	10	0.51	1.44 (0.49-4.23)
Yes	9	5	0.96	0.96 (0.19-4.74)
Number of prior treatment lines				
0	12	5	0.95	1.06 (0.10-11.76)
1	9	1	0.96	2.47 (0.82-7.43)
>=2	23	9	0.5	0.50 (0.07-3.62)
Dosage of Disitamab Vedotin				
120mg, d1, q2w	28	12	0.43	1.24 (0.46-3.33)
Others	6	3	0.67	2.21 (0.31-15.82)
Number of treatment cycles				
1-3	15	6	0.86	0.88 (0.23-3.35)
4-8	16	5	0.41	2.04 (0.37-11.22)
>=8	3	4	0.39	60.22 (0.01-668175.28)

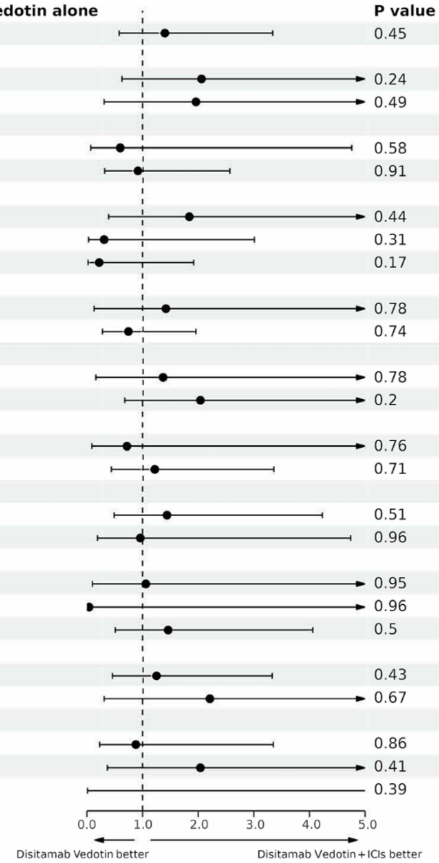

Supplementary Figure 2. Stratified survival analyses of the Disitamab Vedotin combined with ICIs and Disitamab Vedotin cohorts (progression-free survival).

Variables	P value *	HR (95 CI%)
Age		
>=60 vs. <60	0.31	2.07 (0.14 to 31.56)
Sex		
Female vs. Male	0.02*	5.15 (1.25 to 21.16)
Cancer type		
Others vs. Urinary Cancer	0.59	0.36 (0.01 to 15.06)
Pathology		
Adenocarcinoma vs. Urothelial Cancer	0.82	1.53 (0.04 to 61.08)
Metastasis		
Yes vs. No	0.65	0.55 (0.04 to 7.62)
Prior surgery		
Yes vs. No	0.19	0.38 (0.09 to 1.64)
Prior chemotherapy		
Yes vs. No	0.94	0.94 (0.16 to 5.52)
Prior radiotherapy		
Yes vs. No	0.90	1.08 (0.31 to 3.82)
Number of prior treatment lines		
1 vs. 0	0.03*	11.09 (1.35 to 91.38)
>=2 vs. 0	0.40	2.98 (0.23 to 38.17)
Dosage		
<120mg vs. 120mg, d1, q2w	0.83	1.38 (0.07 to 27.61)
Number of treatment cycles		
4-8 vs. 1-3	0.69	0.75 (0.18 to 3.08)
>=8 vs. 1-3	0.18	0.28 (0.05 to 1.80)
Therapy		
Disitamab Vedotin alone vs. Disitamab Vedotin+ICI:	0.29	2.32 (0.49 to 11.02)

*P<0.05

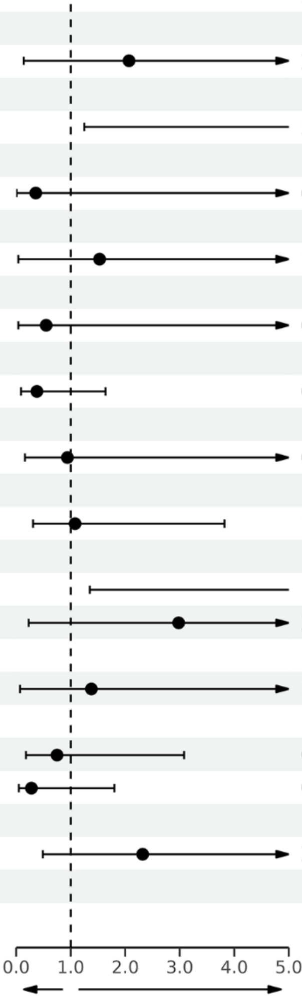

Supplementary Figure 3. Multivariate analysis of the associations between progression-free survival and clinicodemographic variables.
