# Supplementary material for: Efficacy and safety analysis of a HER2-targeting antibody-drug conjugate combined with immune checkpoint inhibitors in solid tumors: a real-world study
Source: Aging (Albany NY). 2023 Dec 22;15(24):15473–88. doi: 10.18632/aging.205382 (PMC10781476; doi:10.18632/aging.205382)
Supplement: Supplementary Tables [file aging-15-205382-s002.pdf]

SUPPLEMENTARY TABLES

Supplementary Table 1. ICIs applied in this study.

Name of ICIs	Target	No. of treated patients	%
Toripalimab	PD-1	14	47.0
Tislelizumab		6	17.6
Camrelizumab		3	8.8
Pembrolizumab		2	5.9
Serplulimab		3	8.8
Sintilimab		2	5.9
Atezolizumab	PD-L1	1	2.9
Envofolimab		1	2.9
Cadonilimab	PD-1+CTL-4	2	5.9
**All**		**34**	**100**

Supplementary Table 2. Best overall tumor responses in the urinary cancer populations.

Populations	Total no.	PD, no. (%)	SD, no. (%)	PR, no. (%)	ORR (95% CI)	DCR (95% CI)
**Treatment**						
Disitamab Vedotin + ICIs	19	6 (31.6)	6 (31.6)	7 (36.8)	36.8 (17.2-61.4)	68.4 (43.5-86.4)
Disitamab Vedotin alone	7	2 (28.6)	3 (42.9)	2 (28.6)	28.6 (5.1-69.7)	57.1 (20.2-88.2)
**HER2 expression**						
0-1+	5	2 (40.0)	1 (20.0)	2 (40.0)	40.0 (7.3-83.0)	60.0 (17.0-92.7)
2-3+	18	4 (22.2)	8 (44.4)	6 (33.3)	33.3 (14.3-58.8)	55.6 (31.4-77.6)
Unknown	3	2 (66.7)	0 (0.0)	1 (33.3)	33.3 (1.8-87.5)	33.3 (1.8-87.5)
**All populations**	**26**	**8 (30.8)**	**9 (34.6)**	**9 (34.6)**	**34.6 (18.0-55.6)**	**69.2 (48.1-85.0)**

Supplementary Table 3. Detailed information on 12 patients with rare tumors.

Patient number	Combined ICIs	Age	Sex	Cancer type	Therapy line	Her2 expression level	Best response	PFS (months)	OS (months)
1	PD-1	56	Female	Cholangiocarcinoma	1	1	SD	4*	4*
2	PD-1	31	Male	Scroti carcinoma	3	2	PR	8*	8*
3	PD-L1	69	Female	Lung cancer	6	Uk	PD	6	13*
4	PD-1	47	Male	Salivary duct carcinoma	7	2	PR	7	11*
5	PD-1	51	Male	Extramammary Paget disease	2	Uk	PR	11*	11*
6	PD-1	28	Female	Pancreatic cancer	3	1	SD	14*	14*
7	PD-1	62	Male	Salivary duct carcinoma	2	2	PR	15*	15*
8	PD-1	57	Female	Cervical Cancer	3	Uk	PD	7	20*
9	PD-1	57	Male	Esophageal cancer	2	3	PR	13*	13*
10	-	56	Female	Ampullary carcinoma	3	2	PD	3	9*
11	PD-1	41	Female	Ovarian cancer	2	Uk	SD	21*	21*
12	PD-1	60	Male	Scroti carcinoma	3	2	PR	5*	5*

*Events did not happen until the end of the follow-up.

Abbreviation: PFS, progression-free survival; OS, overall survival; Uk, unknown; PR, partial response; PD, progression disease; SD, stable disease.
